# Clinical and Biomechanical Predictors of Forefoot Plantar Pressure in Individuals with Type 2 Diabetes and Peripheral Neuropathy

**DOI:** 10.3390/medicina62091701

**Published:** 2026-09-05

**Authors:** Cansu Koltak Altan, Yasin Yurt

**Affiliations:** Department of Physiotherapy and Rehabilitation, Faculty of Health Sciences, Eastern Mediterranean University, Mersin 99628, Türkiye; cansu.koltak@emu.edu.tr

**Keywords:** type 2 diabetes, peripheral neuropathy, plantar pressure, ankle stiffness

## Abstract

*Background and Objectives*: Abnormal plantar pressure distribution is an important biomechanical risk factor for diabetic foot ulceration in individuals with type 2 diabetes (T2D) and peripheral neuropathy. Although several factors have been linked to increased forefoot plantar pressure, factors associated with the forefoot-to-rearfoot pressure ratio remain unclear. This study aimed to investigate the clinical factors associated with the forefoot-to-rearfoot pressure ratio and peak forefoot pressure in this population. *Materials and Methods*: This study was a cross-sectional analysis of 84 individuals with T2D and peripheral neuropathy enrolled in a randomized controlled trial. Vibration perception threshold (VPT), passive ankle dorsiflexion range of motion, ankle stiffness, plantar pressure distribution, body mass index (BMI), HbA1c, duration of diabetes, and foot deformities were assessed. Multiple linear regression analyses were performed to identify factors associated with plantar pressure outcomes. *Results*: The model for forefoot-to-rearfoot pressure ratio explained 51.2% of the variance (adjusted R^2^ = 0.467); ankle stiffness (β = 0.505, *p* < 0.001), foot deformity (β = 0.194, *p* = 0.030), and ankle dorsiflexion range of motion (β = −0.363, *p* = 0.001) were independent predictors. The model for peak forefoot pressure explained 31.4% of the variance (adjusted R^2^ = 0.251); ankle stiffness (β = 0.337, *p* = 0.002), foot deformity (β = 0.278, *p* = 0.009), and BMI (β = 0.226, *p* = 0.029) were independent predictors. HbA1c, diabetes duration, and VPT were not significant in either model. *Conclusions*: In individuals with T2D and peripheral neuropathy, plantar pressure distribution, particularly forefoot loading, appears to be more closely related to biomechanical and structural factors than to metabolic indicators. These findings suggest that assessment of ankle stiffness, joint mobility, and foot posture may provide clinically relevant information for identifying biomechanical factors associated with unfavorable plantar loading in individuals with T2D and peripheral neuropathy.

## 1. Introduction

Diabetic foot ulcers, one of the chronic complications of type 2 diabetes (T2D), are a common problem affecting approximately 15–25% of individuals and resulting in amputation in 20% of cases [1]. Peripheral neuropathy and vasculopathy, together with loss of protective sensation, intrinsic muscle atrophy, toe and foot deformities, altered plantar pressure distribution, and reduced tissue elasticity, have been implicated in the pathogenesis of diabetic foot ulcers [2,3].

In T2D, prolonged hyperglycemia leads to non-enzymatic glycation of collagen tissue, resulting in increased stiffness and hypomobility in the plantar tissues and ankle [4,5]. Previous evidence suggests that impaired foot and ankle mobility may alter the rocker mechanism during gait and contribute to increased forefoot plantar loading, a region commonly affected by diabetic foot ulceration [6,7]. Caselli et al. showed that peak forefoot pressure and the forefoot-to-rearfoot pressure ratio increase the risk of diabetic foot ulcers [8]. Therefore, monitoring and preventing increased plantar pressure, which creates a traumatic basis for ulcer formation, is one of the important protective approaches in the diabetic foot [9,10].

Studies have shown that altered plantar loading in T2D arises as a result of various metabolic and biomechanical factors. Some studies investigating increased peak pressure in the forefoot have found this increase to be associated with high body mass index (BMI) [11], disease duration [12], the presence and severity of neuropathy [13,14,15], toe and foot deformities [11,16,17], reduced ankle mobility [17], and increased plantar fascia stiffness [17,18]. Although studies have reported increased distal soft tissue stiffness in individuals with diabetes, its relationship with plantar pressure distribution has received comparatively limited attention. Couppé et al. showed that Achilles tendon stiffness and the forefoot-to-rearfoot pressure ratio were higher in individuals with diabetes than in age-matched healthy controls [4]. Giacomozzi et al. also demonstrated that the Achilles tendon and plantar fascia were thicker in individuals with diabetes than in healthy subjects, and that the reduction in joint mobility associated with increased thickness could explain a substantial proportion of the increased loading under the metatarsal heads [6].

Importantly, range of motion (ROM) and stiffness reflect distinct biomechanical properties: ROM quantifies the available angular excursion of the joint, whereas stiffness reflects the resistance of the joint and surrounding soft tissues to movement within that range, and the two do not necessarily change in parallel [19]. Consequently, two individuals with comparable ROM may exhibit markedly different stiffness values, indicating that ROM alone may not fully characterize the mechanical behavior of the foot and ankle complex.

Most previous studies examining clinical factors associated with forefoot plantar pressure have included ankle range of motion in their multivariable analyses, whereas ankle stiffness has not been evaluated as an independent predictor. In addition, no comprehensive predictive study has been conducted to examine the factors affecting the forefoot-to-rearfoot pressure ratio, which is another established risk factor for diabetic foot ulcers. Therefore, the aim of the present study was to investigate the clinical factors independently associated with each of two plantar pressure outcomes: the forefoot-to-rearfoot pressure ratio and peak forefoot pressure in individuals with type 2 diabetes and peripheral neuropathy, including ankle stiffness in the analysis.

## 2. Materials and Methods

### 2.1. Study Design and Participants

This study was conducted as a baseline cross-sectional analysis of all participants enrolled between November 2022 and November 2024 in a randomized controlled trial performed in an outpatient clinic, investigating the effects of different exercise approaches on plantar pressure and ankle stiffness in individuals with type 2 diabetes and peripheral neuropathy. Only pre-randomization baseline data were analyzed; therefore, treatment allocation did not influence the present cross-sectional analysis.

The inclusion criteria were a vibration perception threshold (VPT) ≥25 V measured at the hallux, which was used as the cutoff for peripheral neuropathy, and the ability to walk at least 100 m independently. The exclusion criteria were the presence of an active foot ulcer, history of amputation, history of lower extremity surgery, presence of Charcot foot deformity, or participation in a regular exercise program within the previous 3 months. The study was approved by the Scientific Research and Publication Ethics Committee of Eastern Mediterranean University (approval no. ETK00-2021-0195), and all participants who agreed to participate signed a written informed consent form confirming their voluntary participation.

A total of 118 individuals with type 2 diabetes were assessed for eligibility. Of these, 87 met the eligibility criteria and were enrolled in the study. Three participants withdrew before completing the baseline measurements; therefore, 84 participants were included in the final analysis.

Based on the relevant literature, seven variables were included as potential predictors: duration of diabetes, BMI, HbA1c, VPT, passive ankle dorsiflexion range, ankle stiffness, and foot deformities (pronated/supinated). As the present study was a secondary cross-sectional analysis of baseline data from the parent randomized controlled trial, no separate a priori sample size calculation was performed for the regression analyses. All participants with complete baseline data were included in the present analysis.

### 2.2. Outcome Measures

After recording participants’ age, duration of diabetes, and HbA1c values obtained from biochemical analysis, body weight and height measurements were documented. All limb-specific assessments, including VPT, ankle range of motion, ankle stiffness, foot posture, and plantar pressure, were performed on the dominant lower limb, and only data from this limb were included in the analyses.

#### 2.2.1. Vibration Perception Threshold

Participants’ VPTs were assessed at hallux using a biothesiometer (Vibrometer VPT^®^, Diabetic Foot Care, Chennai, India). Before the assessment, the vibration sensation to be perceived was demonstrated by applying the stimulus to the participants’ palms. The probe of the device was applied vertically to the relevant points with constant pressure, and the voltage was increased at a rate of 1 mV/s. The value at which the participant first perceived the vibration was recorded in volts. VPT was assessed at the hallux, first, third, and fifth metatarsal heads, midfoot, and heel. Each site was assessed three times, and the mean value was calculated for each site [20]. For the statistical analyses, the overall mean VPT across all assessed plantar sites was used.

#### 2.2.2. Ankle Range of Motion and Stiffness

Passive ankle dorsiflexion range of motion and dorsiflexion stiffness were assessed using an isokinetic dynamometer (Humac Norm, Computer Sports Medicine Inc., Stoughton, MA, USA). The isokinetic dynamometer was calibrated according to the manufacturer’s instructions before each testing session. Participants were positioned prone with the knee in extension and the ankle in the subtalar neutral position, and the thigh and foot were stabilized with Velcro straps. In this position, the maximum passive ankle dorsiflexion angle was first recorded by pushing the dynamometer arm into dorsiflexion.

For the assessment of passive dorsiflexion stiffness, the continuous passive motion mode of the dynamometer was used. An angular velocity of 60°/s was applied in order to reflect walking-related angular velocity [21]. Gravity correction was applied to eliminate the effect of foot weight during the measurement. Before the measurement, five repetitions of passive ankle motion were performed. Gastrocnemius muscle relaxation was confirmed during the measurements using surface electromyographic biofeedback. The continuous passive motion range was set from 5° of plantar flexion to the maximum dorsiflexion limit. The movement was repeated three times, and the torque-angle curve was obtained. The dorsiflexion stiffness value was calculated using the torque difference between the neutral ankle position and maximum dorsiflexion, together with the range of motion, according to the following formula [22]:

$$Stiffness\;formula\;(\frac{Nm}{rad})=\frac{\max\;dorsilexion\;torque-neutral\;ankle\;torque}{passive\;dorsilexion\;range}$$

In the reliability analysis performed before the study, the intrarater and interrater Intraclass Correlation Coefficient (ICC) values for stiffness assessment were 0.947 and 0.931, respectively (95% CI).

#### 2.2.3. Plantar Pressure

For plantar pressure assessment, a fixed pedobarographic platform (MediLogic Platform Basic, Schönefeld, Germany) embedded in a 6-m walkway was used. After one familiarization trial, the two-step protocol recommended for individuals with diabetes was applied [23]. On the obtained pressure distribution map, the plantar surface was divided into three anatomical regions representing the forefoot, midfoot, and rearfoot, and the peak pressure values for each region were recorded in N/cm^2^. The forefoot-to-rearfoot pressure ratio was then calculated by dividing the values obtained from these regions.

#### 2.2.4. Foot Posture

Foot posture was assessed using the six-item Foot Posture Index (FPI-6). According to the established FPI-6 classification, scores of 6 or higher indicated pronation, and scores of −1 or lower indicated supination of foot [24].

### 2.3. Statistical Analysis

All statistical analyses were performed using SPSS, Version 18.0 (SPSS Inc., Chicago, IL, USA). Continuous variables were assessed using descriptive statistics and presented as mean ± standard deviation ($\bar{x}$ ± SD), whereas categorical variables were expressed as frequencies and percentages [n (%)].

Associations between continuous variables were evaluated using Pearson correlation analysis. Multiple linear regression analyses were conducted to identify independent predictors of the forefoot-to-rearfoot plantar pressure ratio and peak forefoot plantar pressure. Variables considered clinically and biomechanically relevant, including BMI, duration of diabetes, HbA1c, VPT, passive ankle dorsiflexion range, ankle stiffness, and foot deformity, were entered into the regression models. Foot deformity was entered into the regression models as a binary variable. Participants with normal foot posture were coded as 0, whereas those with either pronated or supinated were coded as 1, representing the presence of foot deformity.

Model assumptions were evaluated prior to analysis, including linearity, normality of residuals, independence of errors, and multicollinearity. Multicollinearity was assessed using tolerance values and variance inflation factors (VIFs). Regression results were reported as unstandardized coefficients (B), standard errors (SEs), standardized coefficients (β), 95% confidence intervals, and *p*-values. Model fit was evaluated using R^2^ and adjusted R^2^ values. Significance level was set at *p* < 0.05.

## 3. Results

The study included a total of 84 individuals with type 2 diabetes (49 females and 35 males). Participant demographics and clinical characteristics, including age, gender, BMI, duration of diabetes, HbA1c, VPT, foot posture, ankle dorsiflexion range of motion, and ankle dorsiflexion stiffness, are summarized in Table 1. In addition, forefoot and rearfoot peak plantar pressures and the forefoot-to-rearfoot pressure ratio are also presented (Table 1).

### 3.1. Forefoot-to-Rearfoot Pressure Ratio

A multiple linear regression model was constructed to identify factors associated with the forefoot/rearfoot plantar pressure ratio, explaining 51.2% of the variance in the dependent variable (R^2^ = 0.512; adjusted R^2^ = 0.467). In this model, higher stiffness (B = 2.220; β = 0.505; *p* < 0.001) and the presence of foot deformity (pronated/supinated) (B = 0.317; β = 0.194; *p* = 0.030) were significantly associated with an increased forefoot-to-rearfoot pressure ratio. In addition, greater ankle dorsiflexion range of motion was significantly associated with a decreased forefoot-to-rearfoot pressure ratio (B = −0.190; β = −0.363; *p* = 0.001). Although duration of diabetes, BMI, HbA1c, and VPT were included in the model, none were independently significant predictors (*p* > 0.05), (Table 2).

### 3.2. Forefoot Peak Plantar Pressure

**Figure 1:**
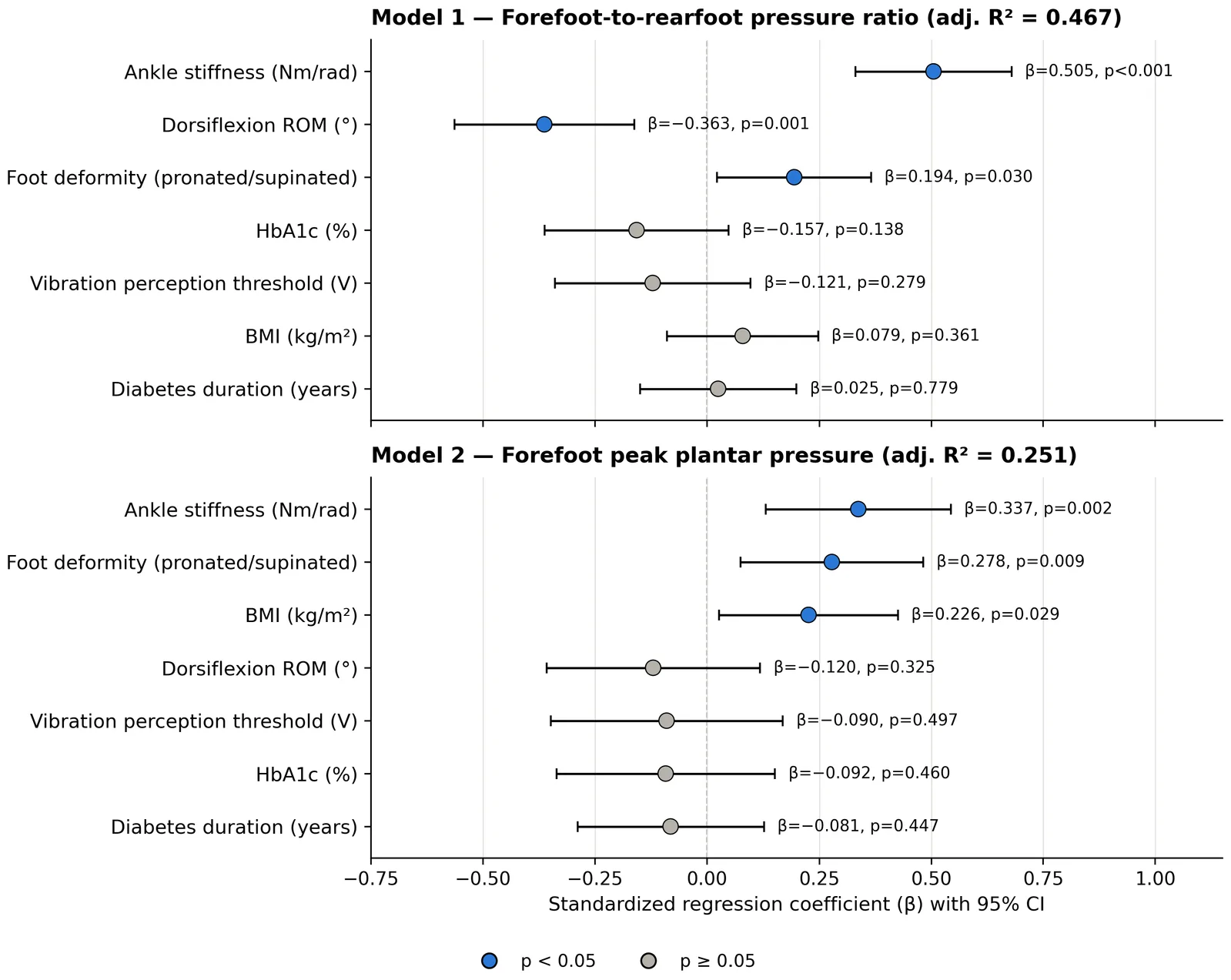
Standardized regression coefficients (β) with 95% confidence intervals for predictors of the forefoot-to-rearfoot plantar pressure ratio (model 1) and peak forefoot plantar pressure (model 2). BMI: Body mass index; ROM: range of motion.; HbA1c: Hemoglobin A1c.

A second multiple linear regression model, with forefoot peak pressure as the dependent variable, explained 31.4% of the variance (R^2^ = 0.314; adjusted R^2^ = 0.251). In this model, BMI (B = 0.679; β = 0.226; *p* = 0.029), stiffness (B = 21.158; β = 0.337; *p* = 0.002), and the presence of foot deformity (pronated/supinated) (B = 6.495; β = 0.278; *p* = 0.009) were identified as independent predictors of forefoot peak pressure. Duration of diabetes, HbA1c, VPT, and ankle dorsiflexion range of motion were not independently associated with forefoot peak pressure (*p* > 0.05), (Table 3). The standardized regression coefficients and 95% confidence intervals for both models are summarized in Figure 1.

## 4. Discussion

The results of the present study indicate that biomechanical and structural characteristics were more consistently associated with plantar pressure distribution than metabolic factors in individuals with T2D and peripheral neuropathy. Ankle stiffness, pronated/supinated foot posture, and ankle dorsiflexion range of motion were independently associated with the forefoot-to-rearfoot pressure ratio, whereas ankle stiffness, pronated/supinated foot posture, and BMI were independently associated with forefoot peak pressure. In contrast, disease duration, HbA1c, and neuropathy severity were not independently associated with either plantar pressure outcome. These findings indicate that biomechanical and structural changes developing in the diabetic foot play an important role in increased plantar pressure in the forefoot.

Ankle stiffness was the strongest predictor of both absolute forefoot peak pressure and the forefoot-to-rearfoot pressure ratio. In diabetes, advanced glycation end products formed by chronic hyperglycemia create irreversible collagen cross-links, altering tendon, ligament, and plantar fascia mechanics and increasing tissue resistance to deformation [19]. This stiffening impairs the shock-absorbing rocker function of the foot, concentrating mechanical loads under the metatarsal heads during gait [6]. Our findings align with Couppé et al., who reported higher Achilles tendon stiffness and forefoot-to-rearfoot pressure ratio in diabetes than in healthy controls [4], and support the clinical relevance emphasized by Caselli et al., who identified this ratio as a predictor of diabetic foot ulceration [8]. Taken together, these findings support an association between altered mechanical properties of the foot and ankle complex and redistribution of plantar loading. Our results extend these observations by showing that ankle stiffness was independently associated with both forefoot peak pressure and the forefoot-to-rearfoot pressure ratio.

Ankle dorsiflexion range of motion affected the forefoot-to-rearfoot pressure ratio but not absolute peak forefoot pressure, possibly by disrupting the rocker movements of the foot and ankle. This is consistent with Mueller et al. and Barn et al., who found only a limited effect of ankle range of motion on forefoot pressure [16,17], and with Turner et al., who reported no association between dorsiflexion range of motion and peak forefoot pressure despite reduced passive range of motion in diabetes [25]. Similarly, Orendurff et al. concluded that ankle equinus deformity explains only a small proportion of increased forefoot pressure [26]. Although reduced joint mobility and increased stiffness often co-occur in type 2 diabetes, they are not equivalent constructs [19]. Stiffness reflects resistance to movement within the available range, so two individuals with similar range of motion may differ substantially in tissue stiffness. This distinction likely explains why ankle stiffness emerged as the more sensitive predictor of forefoot pressure in our study. Clinically, both joint mobility and the stiffness of structures limiting dorsiflexion should be assessed when planning preventive strategies. In our parent randomized controlled trial, adding targeted foot and ankle exercises produced the greatest reductions in ankle stiffness and pressure ratio after 12 weeks [27]. However, changes in plantar pressure were less pronounced than in stiffness itself, suggesting stiffness is a relevant, but not the sole, modifiable determinant of plantar loading.

A pronated/supinated foot was also independently associated with forefoot peak plantar pressure. Weakness of intrinsic foot muscles and reduced mobility from diabetic neuropathy may, over time, alter foot architecture and promote deformity [28]. Such structural alterations may concentrate plantar loading in specific regions and have been associated with an increased risk of ulceration [29]. In contrast to our findings, Tang et al. and Barn et al. did not identify pes planus/cavus as predictors of forefoot pressure in diabetes [11,16]. Differences in methods used to characterize foot posture, and the distribution of specific foot posture abnormalities may partly account for the inconsistent findings across studies. In the present study, pronated and supinated foot postures were combined into a single foot posture category. Because these configurations may produce different regional loading patterns, this approach may have obscured posture-specific associations with forefoot pressure. Previous work has instead highlighted hallux valgus and hammer/claw toe deformities as important predictors of forefoot pressure [11,16,17]; our foot posture effect may partly reflect unassessed accompanying toe deformities. Supporting this interpretation, Ledoux et al. reported an association of pes planus with hallux valgus and of pes cavus with hammer toe [30]. They also found a higher ulcer risk in the presence of fixed hammer/claw toes and hallux limitus. Incorporating toe deformity and deformity severity, rather than presence/absence alone, may improve future models predicting forefoot pressure in T2D.

BMI independently predicted peak forefoot pressure, consistent with a direct mechanical effect of body weight on plantar loading and increased ground reaction forces during gait, and in line with Tang et al. [11]. Other studies, however, report no linear BMI and pressure relationship [15,16]; Sutkowska et al. found increased forefoot pressure only below a BMI of 35, with loading shifting to the midfoot and lateral foot at higher BMI [31]. As our participants’ mean BMI was below 35, this may explain the association observed here. Notably, BMI was not associated with the forefoot-to-rearfoot ratio, suggesting that increased body weight raises absolute forefoot loading rather than shifting pressure from rearfoot to forefoot, consistent with the altered gait kinetics reported in obese individuals, including reduced plantar flexion moment [32].

The finding that metabolic variables evaluated in our study, such as disease duration, neuropathy severity, and HbA1c, were not identified as independent determinants of plantar pressure distribution is also noteworthy. Although disease duration and metabolic control are known to be associated with diabetic foot complications, studies specifically linking them to increased forefoot pressure are quite limited. Falzon et al. showed that individuals with a diabetes duration of more than 10 years had higher forefoot plantar pressure than those with a duration of less than 5 years [12]. Guldemond et al. also demonstrated that forefoot pressure was significantly higher in the group with neuropathy [14]. Since the participants in our study had a mean disease duration of 16.9 years and high neuropathy values, the effects of metabolic factors on plantar pressure may have been attenuated. Similar to our population, Barn et al. also showed that disease duration, neuropathy severity, and HbA1c were not predictive of forefoot peak plantar pressure in neuropathic individuals [16]. These findings support the view that increased forefoot plantar pressure in individuals at high risk of ulceration may be explained more by biomechanical factors than by metabolic variables.

This study adds evidence by showing that ankle stiffness is independently associated with both the forefoot-to-rearfoot peak pressure ratio and forefoot peak pressure in individuals with T2D and peripheral neuropathy. Few previous studies have incorporated ankle stiffness into analyses of plantar pressure, and, to our knowledge, none have simultaneously evaluated its association with both plantar pressure outcomes in this population. Taken together, our findings suggest that biomechanical and structural characteristics may be more closely associated with altered forefoot loading than the metabolic variables evaluated in this high-risk population. In the prevention of diabetic foot complications, exercises targeting the foot and ankle, as well as interventions aimed at improving mobility and flexibility, may help reduce risk. Indeed, a recent meta-analysis demonstrated the beneficial effects of foot and ankle mobility exercises on ankle range of motion and forefoot plantar pressure [33]. Based on these findings, a multidisciplinary approach including exercise and orthotic interventions aimed at normalizing plantar pressure, in addition to medical treatment, may contribute to the prevention of diabetic foot complications.

Although these findings provide clinically relevant insight into plantar loading in individuals with T2D and peripheral neuropathy, they should be interpreted in light of several methodological limitations. First, because of its cross-sectional design, causal relationships between the variables cannot be established. In addition, plantar pressure measurements were obtained under controlled conditions and loading patterns occurring during daily activities and inside footwear were not evaluated. Pronated and supinated foot postures were also combined into a single abnormal foot posture category in the regression analyses, which may have limited the ability to identify posture-specific associations with plantar loading. Furthermore, since the study group consisted only of individuals with T2D and peripheral neuropathy, the generalizability of the findings to the overall diabetes population is limited. Future prospective studies examining whether the variables identified as predictors of plantar pressure in our study can also predict ulcer development would provide a clearer understanding of the contribution of biomechanical factors to the development of diabetic foot ulcers.

## 5. Conclusions

In individuals with T2D and peripheral neuropathy, biomechanical and structural characteristics were more consistently associated with plantar pressure outcomes than the metabolic and clinical variables evaluated. Ankle stiffness and pronated/supinated foot posture were independently associated with both the forefoot-to-rearfoot pressure ratio and forefoot peak pressure, whereas ankle dorsiflexion range of motion was associated with the forefoot-to-rearfoot pressure ratio and BMI with forefoot peak pressure. These findings highlight the relevance of musculoskeletal and biomechanical assessment in addition to metabolic evaluation in this population. Incorporating measures of ankle stiffness, joint mobility, and foot posture into diabetic foot assessment may help identify mechanical factors associated with unfavorable plantar loading and inform preventive management strategies.

## Figures and Tables

**Table 1:** Descriptive characteristics of participants (n = 84).

Variable	Mean ± SD	Min–Max
Age (years)	59.40 ± 2.90	53–66
BMI (kg/m^2^)	29.94 ± 3.44	23.67–42.22
Duration of diabetes (years)	16.92 ± 3.61	7–25
HbA1c (%)	7.66 ± 0.67	6.6–9.2
Vibration perception threshold (V)	30.34 ± 4.00	21.33–39.16
Ankle dorsiflexion ROM (°)	4.86 ± 1.38	2.0–9.0
Ankle dorsiflexion stiffness (Nm/rad)	0.51 ± 0.16	0.21–0.84
Forefoot peak plantar pressure (N/cm^2^)	44.94 ± 10.34	17.60–64.00
Rearfoot peak plantar pressure (N/cm^2^)	16.67 ± 4.01	12.30–39.90
Forefoot-to-rearfoot pressure ratio	2.85 ± 0.72	1.00–4.88
Gender, n (%)		
Female	49 (58.3)	
Male	35 (41.7)	
Foot Posture, n (%)		
Normal	22 (26.2)	
Pronated	41 (48.8)	
Supinated	21 (25.0)	

BMI, body mass index; ROM, range of motion.

**Table 2:** Multiple linear regression predicting forefoot-to-rearfoot pressure ratio (n = 84).

Predictors	B	SE	β	t	*p*	95% CI	VIF
Constant	3.795	1.032	—	3.677	<0.001 *	1.739–5.851	—
Duration of diabetes (years)	0.005	0.018	0.025	0.282	0.779	−0.031–0.041	1.248
BMI (kg/m^2^)	0.017	0.018	0.079	0.920	0.361	−0.019–0.052	1.140
HbA1c (%)	−0.169	0.113	−0.157	−1.499	0.138	−0.394–0.056	1.716
Ankle dorsiflexion stiffness (Nm/rad)	2.220	0.390	0.505	5.687	<0.001 *	1.442–2.998	1.228
Foot deformity (pronated/supinated)	0.317	0.143	0.194	2.215	0.030 *	0.032–0.603	1.195
Vibration perception threshold (V)	−0.022	0.020	−0.121	−1.089	0.279	−0.062–0.018	1.925
Ankle dorsiflexion ROM (°)	−0.190	0.054	−0.363	−3.547	0.001 *	−0.297–−0.083	1.634

Model summary: R^2^ = 0.512; adjusted R^2^ = 0.467; SEE = 0.528; Durbin–Watson = 1.375. * *p* < 0.05; BMI, body mass index; ROM, range of motion; SE, standard error; CI, confidence interval; VIF, variance inflation factor.

**Table 3:** Multiple linear regression predicting forefoot peak plantar pressure (n = 84).

Predictors	B	SE	β	t	*p*	95% CI	VIF
Constant	35.388	17.482	—	2.024	0.046 *	0.570–70.206	—
Duration of diabetes (years)	−0.233	0.304	−0.081	−0.765	0.447	−0.838–0.373	1.248
BMI (kg/m^2^)	0.679	0.305	0.226	2.223	0.029 *	0.071–1.287	1.140
HbA1c (%)	−1.421	1.913	−0.092	−0.742	0.460	−5.232–2.390	1.716
Ankle dorsiflexion stiffness (Nm/rad)	21.158	6.612	0.337	3.200	0.002 *	7.989–34.327	1.228
Foot deformity (pronated/supinated)	6.495	2.427	0.278	2.676	0.009 *	1.660–11.329	1.195
Vibration perception threshold (V)	−0.233	0.341	−0.090	−0.682	0.497	−0.912–0.446	1.925
Ankle dorsiflexion ROM (°)	−0.900	0.909	−0.120	−0.990	0.325	−2.711–0.911	1.634

Model summary: R^2^ = 0.314; adjusted R^2^ = 0.251; SEE = 8.949; Durbin–Watson = 1.663. * *p* < 0.05; BMI, body mass index; ROM, range of motion; SE, standard error; CI, confidence interval; VIF, variance inflation factor.

## Data Availability

The datasets analyzed in the current study are not publicly available due to patient confidentiality but are available from the corresponding author upon reasonable request.

## Abbreviations

The following abbreviations are used in this manuscript:

T2D	Type 2 diabetes
BMI	Body mass index
ROM	Range of motion
VPT	Vibration perception threshold
ICC	Intraclass correlation coefficient
SD	Standard deviation
SE	Standard error
CI	Confidence interval
VIF	Variance inflation factor
HbA1c	Hemoglobin A1c
